# Identification of a Potential Entry-Fusion Complex Based on Sequence Homology of African Swine Fever and Vaccinia Virus

**DOI:** 10.3390/v16030349

**Published:** 2024-02-23

**Authors:** Jesús Urquiza, Miguel Ángel Cuesta-Geijo, Isabel García-Dorival, Óscar Fernández, Ana del Puerto, José Fernando Díaz, Covadonga Alonso

**Affiliations:** 1 Departamento de Biotecnología, INIA-CSIC, Centro Nacional Instituto Nacional de Investigación y Tecnología Agraria y Alimentaria, Ctra. de la Coruña Km 7.5, 28040 Madrid, Spain; cuesta.miguelangel@inia.csic.es (M.Á.C.-G.); isabel.garcia@inia.csic.es (I.G.-D.); delpuerto.ana@inia.csic.es (A.d.P.); 2 Unidad BICS, Centro de Investigaciones Biológicas Margarita Salas, Consejo Superior de Investigaciones Científicas, Ramiro de Maeztu 9, 28040 Madrid, Spain; oscar.fernandez@cib.csic.es (Ó.F.); fer@cib.csic.es (J.F.D.)

**Keywords:** African swine fever virus, ASFV, NCLDVs, poxviruses, fusion proteins, entry-fusion complex

## Abstract

African swine fever virus (ASFV) belongs to the family of *Asfarviridae*, part of the group of nucleocytoplasmic large DNA viruses (NCLDV). Little is known about the internalization of ASFV in the host cell and the fusion membrane events that take place at early stages of the infection. Poxviruses, also members of the NCLDV and represented by vaccinia virus (VACV), are large, enveloped, double-stranded DNA viruses. Poxviruses were considered unique in having an elaborate entry-fusion complex (EFC) composed of 11 highly conserved proteins integrated into the membrane of mature virions. Recent advances in methodological techniques have again revealed several connections between VACV EFC proteins. In this study, we explored the possibility of an analogous ASFV EFC by identifying ten candidate proteins exhibiting structural similarities with VACV EFC proteins. This could reveal key functions of these ASFV proteins, drawing attention to shared features between the two virus families, suggesting the potential existence of an ASFV entry-fusion complex.

## 1. Introduction

ASFV is the causative agent of African Swine Fever (ASF), a high-mortality hemorrhagic disease affecting swine that is endemic in sub-Saharan Africa. ASF was first discovered in Kenya in 1921, entangling an ancient warthog sylvatic cycle [1]. However, current epidemics outbreaks of ASF in the Caucasus and Russian Federation in 2007 [2] have expanded rapidly over different countries in Europe, Asia, America, and Oceania, driving a huge burden to the global pig industry [3].

African Swine Fever Virus (ASFV) is the only known member of the *Asfarviridae* family, likewise the only known DNA arbovirus. It belongs to the nucleocytoplasmic large DNA viruses (NCLDVs) group. The NCLDV comprises an extensive cluster of eukaryotic viruses including the *Poxviridae*, *Asfarviridae*, and *Phycodnaviridae* families, as well as closely related genera Kaumoebavirus, Pacmanvirus, and Faustovirus [4]. These viruses share double-stranded DNA genomes from about 100 kilobases (kb) to more than 2.5 megabases [5] and a common ancestor [6].

The ASFV genome is approximately 170–193 kb in length and encodes for 150–174 proteins, depending on the virus strains, in which the functions of more than half of the viral genes remain unknown [7].

ASFV infectious cycle starts with viral adsorption and internalization, trafficking into the endocytic pathway [8]. The viral uncoating starts with decapsidation, which should be followed by viral egress from the late endosome (LE). This process, similar for the NCLDV group, is dependent on the acidification of the luminal pH of the endosomes, and other factors [9,10]. Finally, the viral core will be delivered out to the cytoplasm by fusion between the inner viral membrane and the limiting membrane of LE [11]. However, this fusion step and driven cytosolic exit have remained largely obscure for this and other viruses of the NCLDV. ASFV viral proteins (VPs) pE248R and pE199L have been reported to be involved in this membrane fusion step, as suspected because of their sequence similarities to the L1, G9, A16, and J5 fusion proteins of VACV [12]. This might suggest that both viruses can penetrate host cells through similar pathways [12,13].

Recent outbreaks of monkeypox and cowpox infections in humans illustrate that poxviruses remain a global emergency threat [14]. Poxviruses are enveloped, large DNA viruses that also replicate in the cytoplasm of host cells. Depending on the viral strain or host cell type, poxvirus entry depends on the fusion of cellular and viral membranes either at the cell surface or internally following endocytosis [15]. After internalization, the viral core is released into the cytoplasm by a two-step mechanism involving mixing of the cellular and viral lipid membranes, followed by the formation and expansion of a fusion pore between the membrane of the mature virions (MVs) and the endosome membrane [16]. Vaccinia virus (VACV) is the best-described poxvirus due to its use for smallpox virus eradication [17]. The VACV fusion step at the LE membrane is an elaborate process mediated by 11 highly conserved poxviral proteins that form the entry-fusion complex (EFC), 8 of which are conserved in all poxviruses [18]. The EFC is embedded in the membrane of the mature virion and has recently been resolved by super-resolution microscopy [19]. The EFC consists of 11 entry-fusion proteins as mentioned above. Nine of the proteins contain cysteines that are conserved in all poxvirus orthologues. Three proteins (A16, G9, and J5) are homologs derived by duplication. VACV entry-fusion proteins interact in pairs: A28:H2 coimmunoprecipitate together, similar to A16:G9 and G3:L5 with J5. G3:L5, J5:F9, and H2:G9 interactions were revealed by chemical cross-linking of proteins in intact virions [20]. Each of these EFC proteins also interact with protein O3, a small protein that may be repeated in the complex. O3 may stabilize the flat two-dimensional structure of the EFC, given through the transmembrane domain present in all EFC proteins [21]. It has been recently revealed that the deletion of O3 can be partially compensated by mutations in the hydrophobic domains of J5 and F9 [22].

Recent studies have highlighted the conservation of entry-fusion proteins within the Poxvirus family and more broadly within the NCLDV family [10,23,24]. To date, only two ASFV proteins have been found to share certain homology with VACV EFC proteins. In this study, we propose the inclusion of some previously undescribed ASFV proteins as potential components of an ASFV EFC. This complex would play a crucial role in mediating the fusion process between the inner viral membrane and the limiting membrane of the LE, based on their similarity with VACV EFC proteins. Comprehensive whole-genome alignments were performed between VACV EFC proteins and the complete ASFV genome and proteome. This analysis identified specific ASFV proteins that may contribute to a potential, yet simplified ASFV entry-fusion complex important for the membrane fusion and subsequent release into the cytoplasm.

## 2. Materials and Methods

### 2.1. Plasmid Constructs

Part of the methodology used in this part of the study has been described previously [25]. To generate plasmids expressing the ASFV Georgia MGF360-15R, p34 (CP2475L), MGF360-16R, MGF360-2L, M448R, and F165, a codon-optimized cDNA sequence for the ORF of the different ASFV Georgia proteins (NCBI reference sequence number: NC_044959.2) was cloned into the pEGFP-C1 (from GeneArt, Thermo Fisher Scientific Waltham, MA, USA). After cloning, the sequence of the plasmids was confirmed by sequencing (Gene Art, Thermo Fisher Scientific).

To generate the ASFV Georgia-E248R or E199L with three copies of the N-terminal HA tag (3xHA E248R and 3xHA E199L), a codon-optimized cDNA sequence for the ORF of the ASFV Georgia (NCBI reference sequence number: NC_044959.2) was cloned into the pKH3 plasmid (Addgene plasmid #12555). After cloning, the sequence of 3xHA E248R and 3xHA E199L plasmids was confirmed by sequencing (Gene Art, Thermo Fisher Scientific).

To generate the ASFV BA71-I215L with 3 copies of C-terminal flag tag (I215L 3xFlag), a codon-optimized cDNA sequence for the ORF of the ASFV BA71 (NCBI reference sequence number: NC_001659) was cloned into the pcDNA3.1 plasmid. After cloning, the sequence of the plasmids I215L 3xFlag was confirmed by sequencing (Gene Art, Thermo Fisher Scientific).

### 2.2. Cell Culture and Transfections

Human embryonic kidney cells (HEK293T) were cultured at 37 °C in a 5% CO_2_ atmosphere culture in complete Dulbecco’s Modified Eagle Medium (DMEM) supplemented with 5 or 10% heat-inactivated fetal bovine serum (FBS) respectively, 1% penicillin-streptomycin (P/S) and 1% Glutamax (Gibco, Gaithersburg, MD, USA).

For transfection of HEK 293T cells, two 60 mm plates were seeded with 3.5 × 10^6^ cells each 24 h before transfection in DMEM complete medium as described above. Before transfection, the medium was changed to DMEM complete medium with 2% FBS. Cotransfection of 2 μg of plasmids HA E248R, HA E199L, I215L Flag, and EGFP plasmids in pairs for each 60 mm dish was then performed using Lipofectamine 2000 (Thermo Fisher Scientific), according to the manufacturer’s instructions. Twenty-four hours after transfection, cells were harvested, lysed, and immunoprecipitated using a GFP-Trap kit (Chromotek, Planegg-Martinsried, Germany).

### 2.3. Immunofluorescence

To confirm the expression of the EGFP-tagged plasmids, each plasmid was transfected into Heck293T and seeded onto 12 mm glass coverslips. Nuclei were detected with TOPRO3. The coverslips were mounted on glass slides using ProLong Gold (Thermo Fisher Scientific; Waltham, MA, USA). Overexpressed plasmids in 293T cells were imaged using a TCS SPE confocal microscope (Leica, Wetzlar, Germany) with a 63× oil immersion objective. Image acquisition was performed using Leica Application Suite Advanced Fluorescence Software (LAS AF v3.5.2.18963).

### 2.4. Alignments and Sequence Similarity

SIM Alignment Tool for Protein Sequences from Expasy “https://web.expasy.org/sim/” (accessed on 10 February 2023) and Geneious Prime^®^ v2022.1.1 were used to perform the whole genome alignments.

NCBI reference sequence NC_001659.2 was used for the ASFV Ba71V reference genome, NCBI reference sequence NC_044959.2 was selected for the ASFV Georgia 2007/1 reference genome, and GenBank KM111295.1 was used for the ASFV Kenya reference genome. The NCBI Reference Sequence NC_006998.1 was used for the vaccinia virus strain reference genome and NC_002520.1 was selected for the *Amsacta moorei* Betaentomopoxvirus complete genome.

For the sequence alignments provided in Supplementary Figure S2 and Supplementary Table S1, Geneious Alignment with Blosum62 Needleman-Wunsch matrix was used, with a threshold value of 3. SIM from Expasy (SIB Swiss Institute of Bioinformatics, Lausanne, Switzerland) was used for Supplementary Figure S1, using the default parameters of the Blosum62 matrix. Using the default parameters of Geneious alignment, amino acids aligned from different protein species that equal or exceed the specified threshold of 3 with a Blosum62 Needleman–Wunsch matrix, implying 100% similarity, are shown in red. Red represents 100% amino acid identity, while light red represents an 80–90% range in physicochemical similarity properties between the amino acids of the two proteins.

### 2.5. EGFP Coimmunoprecipitations

This part of the methodology was similar to that used in [23], where the coimmunoprecipitations (IP) of EGFP BA71 ASFV proteins with other cellular proteins were performed using a GFP-Trap^®^_A (Chromotek , Planegg-Martinsried, Germany). For IPs, the cell pellet was resuspended in 200 μL of lysis buffer (10 mM Tris HCl pH 7.5; 150 mM NaCl; 0.5 mM EDTA; 0.5% NP40) and then incubated on ice for 30 min. The lysate was then clarified by centrifugation at 14,000× *g* and diluted five-fold with dilution buffer (10 mM Tris HCl pH 7.5; 150 mM NaCl; 0.5 mM EDTA). GFP-Trap agarose beads were equilibrated with ice-cold dilution buffer and then incubated with diluted cell lysate overnight at 4 °C on a rotator, followed by centrifugation at 2500× *g* for 2 min. The bead pellet was washed twice with wash buffer (10 mM Tris-HCl pH 7.5; 150 mM NaCl; 0.5 mM EDTA). After removal of the wash buffer, the beads were resuspended in 100 μL of sample buffer Laemmli 2X concentrate (Sigma-Aldrich/Merck; Darmstadt, Germany) and boiled at 95 °C for 10 min to elute the bound proteins. Immunoprecipitation buffers were supplemented with Halt Protease Inhibitor Cocktail EDTA-Free (Thermo Fisher Scientific).

### 2.6. Western Blot Analysis and Antibodies

Coimmunoprecipitation samples in Laemmli buffer were resolved on SDS-PAGE in Mini-PROTEAN TGX 4–20% gradient gels (BioRad, Hercules, CA, USA). The gels were then transferred to a nitrocellulose membrane (Bio-Rad) using the Trans-Blot Turbo Transfect Pack (Bio-Rad) and the Trans-Blot Turbo System (Bio-Rad), and detected by Western blot analysis using the appropriate antibodies: anti-GFP (Santa Cruz Biotechnology, Dallas, TX, USA), anti-Flag (Sigma), anti-HA (Invitrogen, Thermo Fisher Scientific; Waltham, MA, USA), and anti-GAPDH (Abcam, Cambridge, United Kingdom) as loading control. Anti-mouse IgG (GE Healthcare, Chicago, IL, USA) or anti-rabbit IgG (Bio-Rad) conjugated to horseradish peroxidase was used the secondary antibody at a dilution of 1:5000. Finally, the bands obtained after development with ECL reagent were detected on a Molecular Imager Chemidoc XRSplus imaging system.

### 2.7. AlphaFold2 3D Structural Protein Modelization

The AlphaFold2 v2.1.2 (AF2) [26] program was used to generate the 3D protein models of the ASFV Georgia proteins E248R, E199L, MGF360-15R, MGF360-16R, p34, F165R, M448R, MGF360-2 L, G1340L, and p1192R; and the EFC 11 VACV proteins F9L, L1R, H2R, A28L, A21L, J5L, L5R, G3L, A16L, G9R, and O3L, and A26L which binds A16L and G9R in the VACV complex. The NCBI reference sequence NC_044959.2 was used for the ASFV Georgia reference genome, and NC_006998.1 was selected for the vaccinia virus reference genome.

AF2 ran on a high-performance computing (HPC) environment based on LADON OS version 7.2Ja. This HPC system was equipped with two A30 NVIDIA GPUs. To obtain accurate and realistic structural predictions, the complete AF2 database, updated up to 1 January 2022, was used. This configuration was kept constant during the prediction of all protein structures presented in this study, ensuring accurate, realistic, and robust structural predictions.

Images were generated using the PyMOL Molecular Graphics System v1.8 [27]. Models with the highest confidence rank value for each protein were selected, comparing the relaxed predictions obtained with AF2.

The multimer display was used for the protein–protein interaction model, taking the two target proteins of ASFV, and selecting the model with the highest confidence score from the 25 different AF2 models for each multimer. AF2 calculates a predicted local distance difference test (pLDDT) score for each residue, which indicates the confidence that the residue C-alpha atom is correctly located relative to nearby residues, indicating the bFactor. The pLDDT score was used as a scoring criterion to validate the structure. For packing evaluation, AF2 calculates a second type of confidence score called the predicted aligned error (PAE) for each pair of residues. The value for different close residues estimates the positional distance error (in Å) when they are aligned with the true structure of the program database. The ChimeraX program v1.7.1 [28] was used to visualize the multimeric structures shown.

The transmembrane domain prediction of the ASFV VPs was performed using the Deep TMHMM program, which provided an accurate prediction of the topology of the proteins [29].

### 2.8. Poxvirus and Asfarvirus Phylogenetic Tree

MUSCLE alignment of DNA polymerase nucleotide sequences was performed before the construction of a neighbor-joining tree with 1000 bootstrap replications using MEGA11: Molecular Evolutionary Genetics Analysis v11 [30], with default parameters. Visualization was also performed using MEGA11 program.

The poxviral and ASFV species are listed in Table 1. The branch confidence number represents a measure of support for the node. The number ranges between 0 and 100, which indicates the number of times virus species are classified in the current arrangement over 100 bootstrap replicates. One thousand bootstrap replicates were used to generate the phylogenetic tree analysis, a method often used in other phylogenetic studies [31].

### 2.9. Alignment and Superposition of Protein Domains with ChimeraX

The superposed protein structure analysis was performed with the Matchmaker display in the ChimeraX v1.7.1 program [28]. VACV EFC proteins were taken as reference structures while their respective ASFV-similar proteins were the structures to match in the program. A chain pairing was made between the best-aligning pair of reference and match structure chains. The alignment was performed with the Needleman–Wunsch algorithm and a Blosum62 matrix, and visualized with the ChimeraX program. Blue was used for ASFV proteins and orange for VACV EFC proteins.

## 3. Results

### 3.1. Search for the Closest Poxviral Species to ASFV by Phylogenetic Analysis

**Figure 1:**
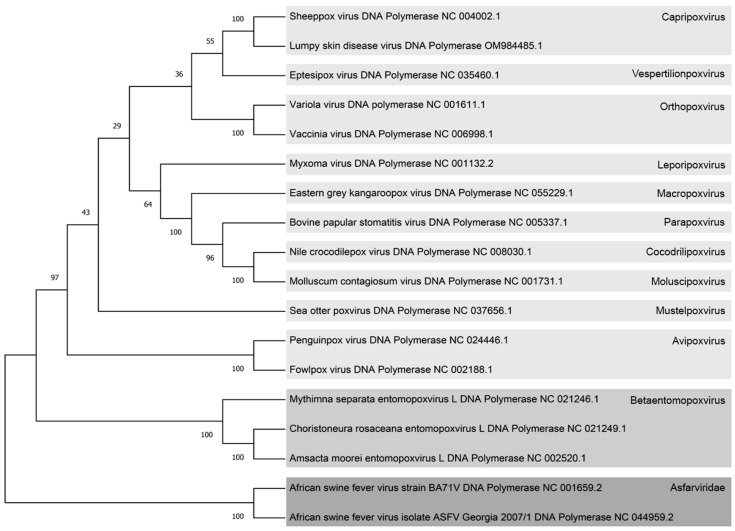
Phylogenetic tree illustrating diverse Poxvirus and Asfarvirus species based on DNA polymerase protein sequences. Each virus is labeled with its respective NCBI reference number, and on the right side, different viral subfamilies are color-coded. The Chordopoxviridae subfamily is represented in light gray, the Entomopoxviridae subfamily in medium light gray, and the Asfarviridae family in dark gray. The confidence branch values are indicated numerically at the intersections of the branches. The gray squares serve to distinguish between the distinct viral subfamilies within the Poxviruses and Asfarviruses.

To identify the closest Poxviral species to ASFV, a phylogenetic analysis of Poxvirus and Asfarvirus species was performed, focusing on the DNA polymerase sequence of each candidate according to the reference genome in Table 1. The analysis involved a MUSCLE alignment followed by the construction of a neighbor-joining tree with 1000 bootstrap replications (Figure 1), as described in Section 2.8 of Materials and Methods.

The resulting tree exhibited two distinct branches, separating the *Asfarviridae* family from the *Poxviridae* family. Within the *Poxviridae* family, the Betaentomopoxvirus subfamily was found to be the closest to the Asfarviruses. Further refinement within the Betaentomopoxvirus subfamily revealed that *Amsacta moorei* Entomopoxvirus (AmEPV) occupied the position of the closest Betaentomopoxvirus to the Asfarvirus family in this phylogenetic tree. This analysis provides valuable insights into the evolutionary relationships between Asfarviruses and Poxviruses, pinpointing specific subfamilies and species that are more closely related phylogenetically.

### 3.2. Genetic Alignment of Similar VACV EFC Proteins in the ASFV Genome through the Closest Phylogenetically Related Poxvirus

In the search for proteins in *Amsacta moorei* Entomopoxvirus (AmEPV), the closest poxvirus to the *Asfarviridae* family that are similar to the entry-fusion complex (EFC) proteins of vaccinia virus (VACV), a thorough search of the entire ASFV genome was conducted. This search involved DNA sequence alignment using the parameters and reference sequences outlined in Section 2.4 of Materials and Methods. The analysis revealed nine AmEPV proteins with similarity to VACV EFC proteins (Supplementary Figure S1A).

Having identified these similar proteins in the complete AmEPV genome, a subsequent search was conducted to identify similarities between these AmEPV proteins and the complete African swine fever virus (ASFV) genome. The alignment analysis resulted in the selection of ten ASFV candidate proteins that exhibited the best nucleotide alignment with the AmEPV proteins (Table 2; Supplementary Figure S1B).

Notably, G3L, O3L, and A26L VACV proteins were not found in the AmEPV whole genome. Therefore, a direct search within the ASFV genome was performed to identify similarities with these VACV EFC proteins.

In the final step of our analysis, the selected ASFV proteins were aligned pairwise with the VACV EFC proteins, as illustrated in Supplementary Figure S2. Identical amino acids are highlighted in red, while similar amino acids are shaded in light red. This alignment was conducted using the previously described parameters outlined in Section 2.4 of Materials and Methods. Gray branches represent similar protein domains. The results, expressed as percentages of identity and similarity data between VACV, AmEPV, and ASFV, are detailed in Supplementary Table S1.

Interestingly, E199L exhibited sequence similarity to three VACV proteins (Supplementary Figure S1A,B). Secondary structure analysis revealed the presence of similar domain regions in the aligned ASFV and VACV proteins (Supplementary Figure S2). However, it should be noted that, due to the differences in sizes between these proteins, the percentage of identity and similarity in their alignments was not particularly high.

### 3.3. Structure and Surface Characterization of VACV- and ASFV-Similar Proteins

In contrast to the relatively well-characterized L1R and F9L [32], other VACV EFC proteins have been only partially crystallized [33,34,35]. Limited information currently exists regarding ASFV proteins implicated in the internalization and LE fusion process. To better understand these early fusion steps, it is imperative to characterize the VACV EFC, and comparisons with ASFV entry proteins would be crucial as an initial investigative strategy. Using the AlphaFold2 program v2.1.2, we modeled the visualization of selected ASFV proteins and VACV EFC proteins.

**Figure 2:**
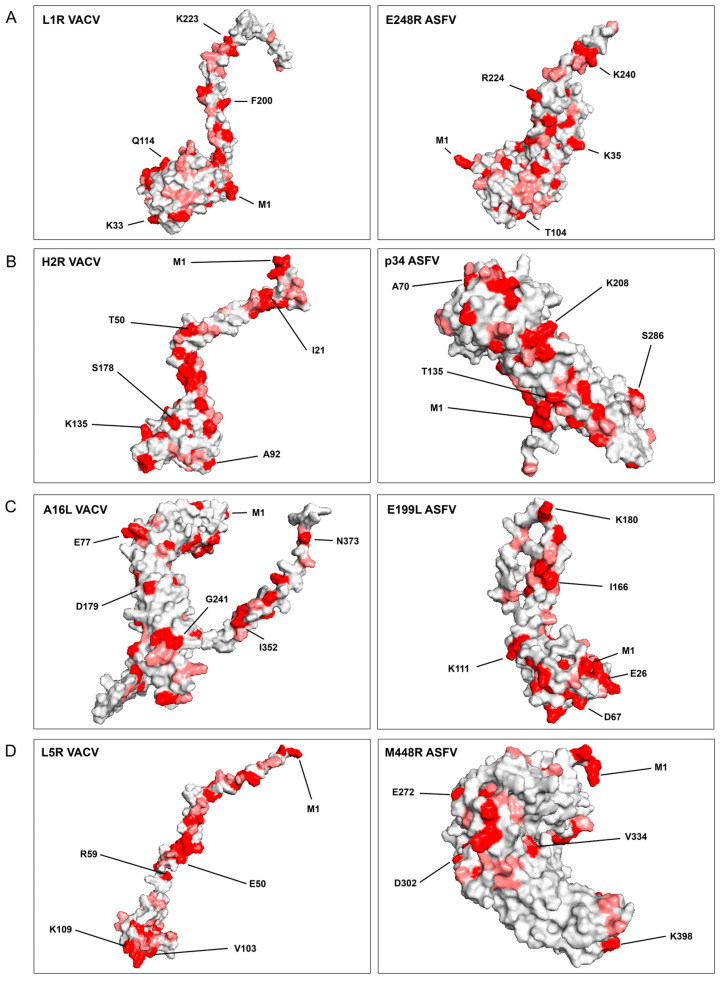
Structure prediction of VACV EFC- and ASFV-similar proteins with the Alphafold2 program. The 3D-accurate computational model presented in Figure 3, generated using the Alphafold2 program, depicts the structural predictions of VACV EFC- and ASFV-similar proteins. Identical amino acids highlighted in red, are shown in pairs between VACV and ASFV proteins. Similar amino acids (with more than 80% similar physicochemical characteristics, as determined by the Blosum62 matrix), are shown in light red. VACV EFC proteins are positioned on the left, while ASFV-similar proteins are on the right. The representative amino acids are numbered and marked by lines in the protein models. Each panel (**A**–**D**) focuses on specific protein pairs: (**A**) L1R of VACV and E248R of ASFV; (**B**) H2R of VACV and p34 of ASFV; (**C**) A16L of VACV and E199L of ASFV; (**D**) L5R of VACV and M448R of ASFV.

The multiple PDBs obtained were ranked based on the accuracy of each model and subsequently visualized using the PyMOL program v1.8 [27]. The top-ranked confidence models were used to characterize the proteins and their corresponding confidence values are presented in Table 3. Prediction values had a pivotal role in the assessment and selection of models, with higher confidence scores being prioritized for further analysis and characterization. To enhance clarity, the surface of the proteins was color-coded under the alignment color key featured in Supplementary Figure S2. This coloring scheme exposed identical amino acids in red and similar amino acids in light red, thus revealing pairs of these residues on the protein surface (Figure 2).

In Figure 2, the VACV EFC proteins are juxtaposed with their potential ASFV homolog, highlighting a remarkable observation. Despite a low linear similarity between the associated ASFV and VACV proteins, a significant number of shared identical and similar amino acids were conspicuously exposed on the surface of the proteins (Figure 2). This observation suggests the plausible existence of similar interactions with cellular target proteins.

### 3.4. Disulfide Bond Conservation between ASFV- and VACV-Similar Proteins and Transmembrane Proteins Domain Analysis

**Figure 3:**
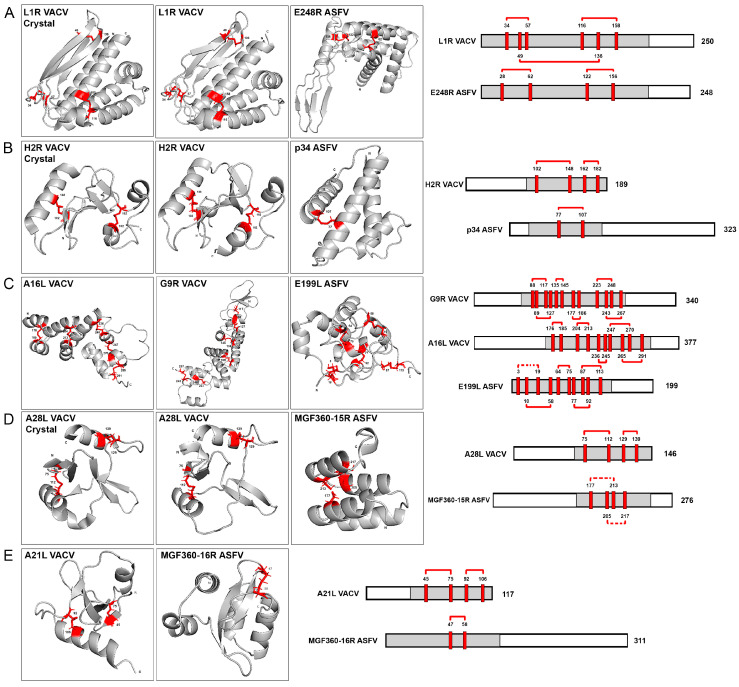
Conservation of cysteine bonds within regions of similar proteins from ASFV and VACV. The gray domains on the left side of the figure represent regions with conserved cysteines in pairs, modeled using the AlphaFold2 (AF2) program. Three available crystal structures are provided in sections (**A**,**B**,**D**) with positions of conserved cysteine residues marked in red. Conserved disulfide bonds are depicted by red solid lines (confirmed in structures predicted by AlphaFold2 or based on X-ray crystallography) or dotted connections (potentially conserved but not predicted by AlphaFold2). The number of amino acids for each protein is indicated at the end of their linear scheme, and the cysteine amino acid numbers are specified at the top of each cysteine residue, corresponding to the crystal structure, AlphaFold2 predictions, and linear protein schemes. The specific pairs of similar proteins follow: (**A**) L1R VACV and E248R ASFV proteins, featuring the crystal structure, AlphaFold2 cysteine models, and a linearized cysteine scheme; (**B**) H2R VACV (crystal and AF2 structures) and p34 ASFV proteins; (**C**) A16L VACV, G9R VACV and E199L ASFV proteins; (**D**) A28L VACV (crystal and AF2 structures) and MGF360-15R ASFV proteins; (**E**) A21L VACV and MGF360-16R ASFV proteins.

In our previous research, we emphasized the significance of the conserved cysteines found in ASFV and Poxvirus EFC proteins [10]. Other studies have also supported the conservation of cysteine bonds in the entry-fusion proteins of various NCLDV members [24]. Our investigation involved a comparison of the disulfide bonding patterns between VACV EFC proteins and their corresponding ASFV-similar proteins. This analysis focused on protein domains highlighted in gray in Figure 3. We examined closely spaced cysteine pairs within these domains that were identified either as established disulfide bonds or others that could potentially form such bonds using structural information obtained from AlphaFold2’s PDBs and recently available crystal structures.

Interestingly, our findings revealed the consistent preservation of disulfide bonding patterns in the structures of poxvirus and ASFV-similar proteins. The L1R VACV structure exhibits three disulfide bonds while the E248R ASFV fusion protein conserves two, as illustrated in Figure 3A. Also in this figure, the L1R crystal structure and the L1R AF2 model are compared. Only very minor differences could be found between both structures. Examining the VACV H2R EFC protein and ASFV p34 protein pair, we observe a consistent conservation pattern. Specifically, one disulfide bond is conserved in p34, while two are maintained in H2R, as illustrated in Figure 3B. Partial crystal structures were recently obtained for VACV H2R protein [35]. This crystal is also compared with the AF2 model for this protein in the Figure 3B. No relevant changes could be observed in the comparison between both structures. This result also supports the AF2 modeling capacity and its accuracy. In the case of VACV protein G9R, six disulfide bonds are conserved, whereas A16L retains five. Additionally, the computational program AF2 predicts five disulfide bonds in E199L, as depicted in Figure 3C. Moving to ASFV protein MGF360-15R and A28L EFC VACV protein, both structures conserved two disulfide bonds, based on the cysteine positions in the AF2 model and recent experimental data, as shown in Figure 3D, also comparing the crystal structure of A28L [34] with the AF2 prediction. Finally, in Figure 3E, the ASFV multigene family 360 protein 16R was found to conserve one disulfide bond aligning with the two conserved in VACV A21L protein (Figure 3E).

These findings would reveal a structurally unique and evolutionarily conserved feature in the entry-fusion proteins of both VACV and ASFV. Turning our attention to the extensively studied transmembrane (TM) domains within VACV EFC proteins, they can be classified in two distinct groups: the N-terminal TM domain proteins (A21, A28, G3, H2, O3, and L5) and the C-terminal TM domain proteins (the homologous A16, G9, and J5; and the proteins L1 and F9). The N-terminal TM proteins exhibit 0–2 intramolecular disulfide bonds, with no sequence similarities among them. Conversely, the C-terminal TM proteins show a higher conservation, with 4–10 conserved disulfide bonds [18].

A similar categorization emerges for the ASFV- and EFC-like proteins. Two groups are identified: eight proteins lacking a TM domain and two proteins featuring C-terminal TM domains (Supplementary Figure S4). The TM domains were predicted using the Deep TMHMM prediction program [29], which distinguishes the C-terminal transmembrane domains (E248R and E199L) from the nontransmembrane domain proteins, among ASFV target proteins. Notably, the ASFV C-terminal domain proteins are potential homologs to the C-terminal domain proteins of VACV, and like their VACV counterparts, conserve 4-10 disulfide bonds. In contrast, non-TM domain ASFV proteins mirror the 0-2 intramolecular disulfide bonds conserved in VACV N-terminal TM domain proteins (Figure 3).

**Figure 4:**
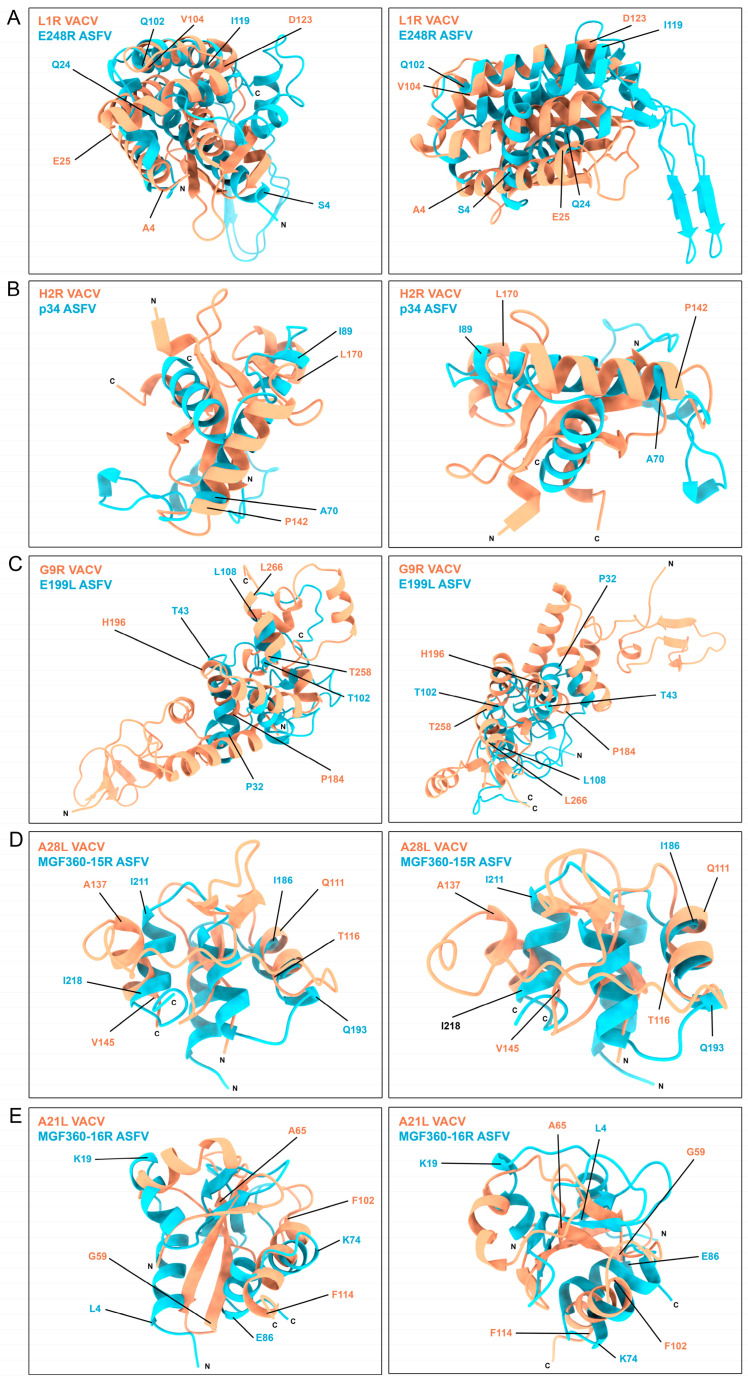
Superposition of VACV- and ASFV-similar protein regions obtained by aligning and overlaying the secondary structure disposition of the compared regions of VACV (orange) and ASFV proteins (blue). The N and C terminal domains of the proteins are annotated for clarity. Relevant regions of the proteins are highlighted and delimited by the corresponding amino acid numbers of the figure, color-coded according to their respective protein color.

A deeper structure comparison was carried out between these similar regions of VACV- and ASFV-similar proteins. Alignments and secondary structure pairing were performed between these protein domains exposed in Figure 3 and Figure 4. These structures were aligned and superposed following the parameters in Section 2.9 of Materials and Methods. In these secondary structure alignments, we could observe the fitting regions of both VACV- and ASFV-similar protein domains. VACV L1R and ASFV E248R proteins share three similar α helix orientations in the disulfide bond region, being completely superposed by the Q102-I119 (E248R) and V104-D123 (L1R) helix (Figure 4A). A similar orientation was found in the superposition of VACV H2R and ASFV p34 structures, where the α helix A70 to I89 (p34) fits on the model of P142 to L170 α helix (H2R) (Figure 4B). A fitting structure seems also to be generated between the VACV G9R and ASFV E199L disulfide bond regions, having similar α helix spatial disposition with different angle orientations (Figure 4C). VACV A28L and ASFV MGF360-15R share two α helix orientations and spatial distribution, as exposed in Figure 4D. The G59-A65 β sheet of VACV A21L follows the orientation of the L4-K19 α helix in ASFV MGF360-16R, similar to the spatial disposition between MGF360-16R K74-E86 and the VACV A21L F102-F114 α helixes (Figure 4E).

These results exhibit the similarities between these disulfide bond shared regions of ASFV and VACV proteins.

### 3.5. Identification of Potential Interactions between Potential ASFV EFC Proteins

The entry-fusion complex of VACV has recently been clarified by different interaction experiments including proximity assays, chemical cross-linking experiments, or immunoprecipitations [21]. ASFV viral proteins (VPs) E248R and E199L have been characterized as fusion proteins for the virus [11,12]. Remarkably, these proteins share structural similarities with several subunits of the poxvirus multiprotein entry/fusion complex (EFC) [10], suggesting their potential inclusion in a putative EFC complex in ASFV.

In an effort to confirm the existence of a potential complex involving the ASFV fusion proteins E248R and E199L and the other identified ASFV candidate proteins, we performed immunoprecipitation assays between the ASFV protein candidates.

For this purpose, reporter protein (EGFP)-tagged were designed for the expression of E248R, E199L, p34, MGF365-15R, MGF360-16R, M448R, MGF360-2L, and F165R ASFV proteins. These proteins were codon-optimized, tagged to EGFP at the N-terminus, and expressed as EGFP-fusion proteins in HEK 293T cells. An empty vector expressing EGFP and the early ASFV protein I215L ubiquitin conjugase were used as controls. All these ASFV VPs were noticed to be highly conserved among ASFV isolates (Supplementary Figure S5). The strategy was based on a high-affinity agarose GFP-coimmunoprecipitation system (GFP-Trap; Chromotek, Chromotek & Proteintech; Planegg-Martinsried, Germany). The ASFV VPs E199L and E248R were cloned in HA tag plasmid, as described in Section 2.1 of Materials and Methods.

**Figure 5:**
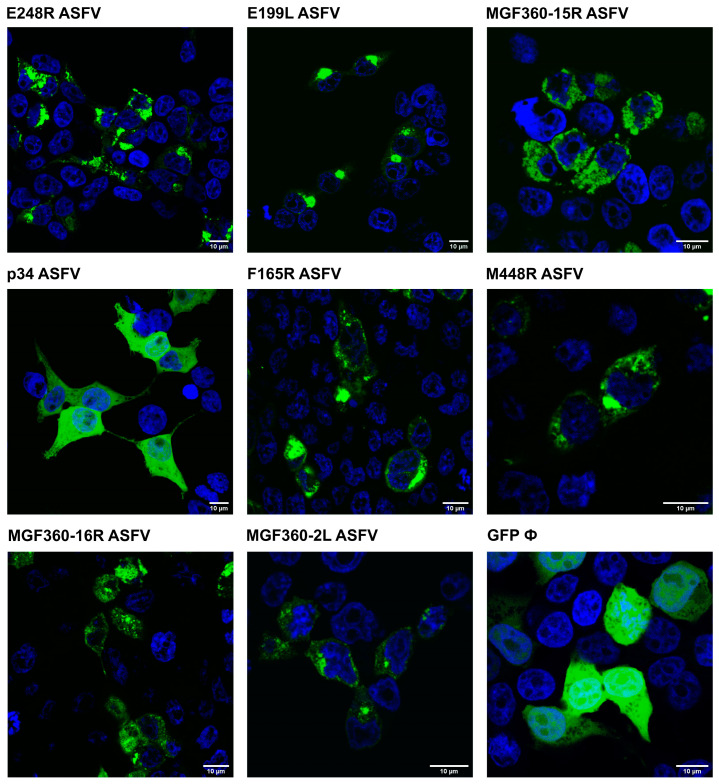
ASFV VP EGFP-tagged expression in cells. ASFV proteins E248R, E199L, MGF360-15R, p34, F165R, M448R, MGF360-16R, MGF360-2L, and EGFP control are transfected onto 293T cells and visualized by confocal fluorescence microscopy. Viral proteins are visualized in green and the nucleus of the cell, stained with TOPRO3, is in blue. A 10 µm scale is indicated in each image.

HEK 293T cells were chosen for this study due to their exceptional transfection efficiency and widespread use as the preferred cell line for protein–protein interaction investigations involving various viruses [36]. This selection is based on the proven effectiveness of HEK 293T cells in enhancing the discrimination between specific and nonspecific interactions with target proteins [10]. The accuracy of the expression of EGFP-tagged viral proteins was verified through immunofluorescence, as depicted in Figure 5.

**Figure 6:**
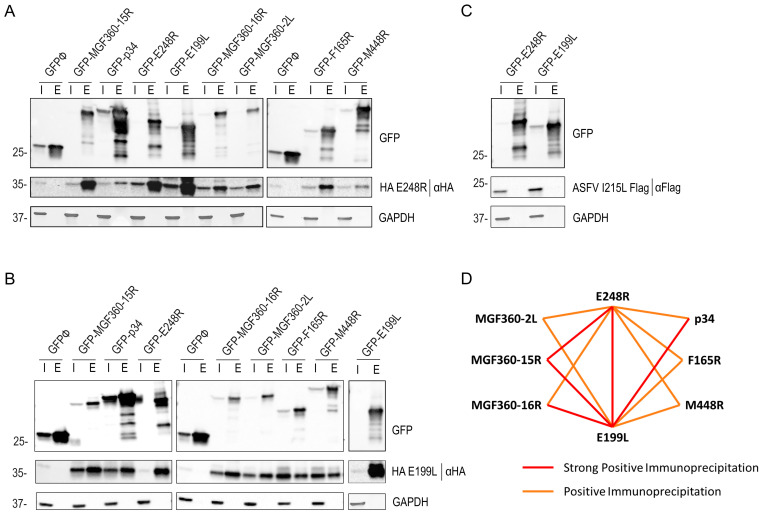
E248R and E199L interactions with viral proteins E248R, E199L, p34, MGF365-15R, MGF360-16R, M448R, MGF360-2L, F165R, and I215L by immunoprecipitation. GFP was immunoprecipitated in lysates from HEK293T cells transfected with EGFP, EGFP E248R, EGFP E199L, EGFP p34, EGFP MGF365-15R, EGFP MGF360-16R, EGFP M448R, EGFP MGF360-2L, and EGFP F165R, and cotransfected with HA E248R (**A**), HA E199L (**B**), or I215L Flag (**C**). Representative immunoblot analysis of cell lysates (I) and GFP immunoprecipitates or elution fraction (E), where GFP, HA, or Flag antibodies were used. GAPDH was used as control. Additional viral protein control I215L ASFV protein was performed (**C**), where the cotransfection of EGFP E248R or EGFP E199L with viral I215L Flag protein was analyzed with GFP, FLAG, and GAPDH antibodies. kDa protein weights are indicated on the left of the images. (**D**) Immunoprecipitation interaction model between ASFV E248R and E199L proteins and the other possible ASFV entry proteins: MGF360-2L, 15R and 16R, F165R, M448R, p34, I215L, and empty vector GFPØ. Two types of interaction are shown: the strong positive immunoprecipitation interaction is in red and the positive immunoprecipitation interaction is in orange, according to the WB triplicate results (Supplementary Figure S6).

Constructs incorporating a reporter protein (EGFP) were specifically designed for the expression of ASFV proteins, namely p34, MGF365-15R, MGF360-16R, M448R, MGF360-2L, E199L, E248R, and F165R. Each EGFP-tagged ASFV viral protein was coexpressed together with HA E248R or HA E199L for 24 h in HEK 293T cells. After cell lysis, immunoprecipitation of the samples was performed using protein G beads and HA-specific monoclonal antibodies [Figure 6]. Expression of HA E248R, HA E199L, and I215LE Flag was then confirmed using anti-HA and anti-Flag antibodies in the whole cell lysates (input) and immunoprecipitated samples (elution) analyzed by WB.

The VPs E248R and E199L tagged with HA were retrieved in the bound samples obtained from Co-IP of the VPs p34, MGF365-15R, MGF360-16R, M448R, MGF360-2L, E199L, E248R, and F165R. The coexpression of EGFP E199L with the HA-tagged E199L and the coexpression of EGFP E248R with the HA-tagged E248R pairs was also performed (Figure 6A,B). Both proteins were retrieved in the bound samples of the Co-IP. In contrast, independent Flag-fused viral protein I215L, used as a negative control with the empty vector EGFP, was not immunoprecipitated, corroborating the specificity of interaction between HA E248R and HA E199L with the other selected ASFV proteins (Figure 6C). Neither HA E248R nor HA E199L was found in the elution of the Co-IP of empty EGFP.

The results obtained from the immunoprecipitation assay revealed a novel and promising potential interaction between ASFV E248R and E199L fusion proteins with the other selected ASFV proteins, which warrants further in-depth investigation. By examining the relative protein abundance in the elution samples compared with each protein expression in the input fractions shown in Figure 6A,B, we have qualitatively outlined an initial interaction scheme. The scheme shown in Figure 6D distinguishes between strong positive immunoprecipitation interactions, positive immunoprecipitation interactions, or negative immunoprecipitation interactions (where there is no expression in the elution fraction) in triplicates (Supplementary Figure S6). These distinctions provide a valuable framework for understanding the nature of the interactions revealed in the study.

### 3.6. Interaction Model between Immunoprecipitated ASFV Proteins with AlphaFold2

**Figure 7:**
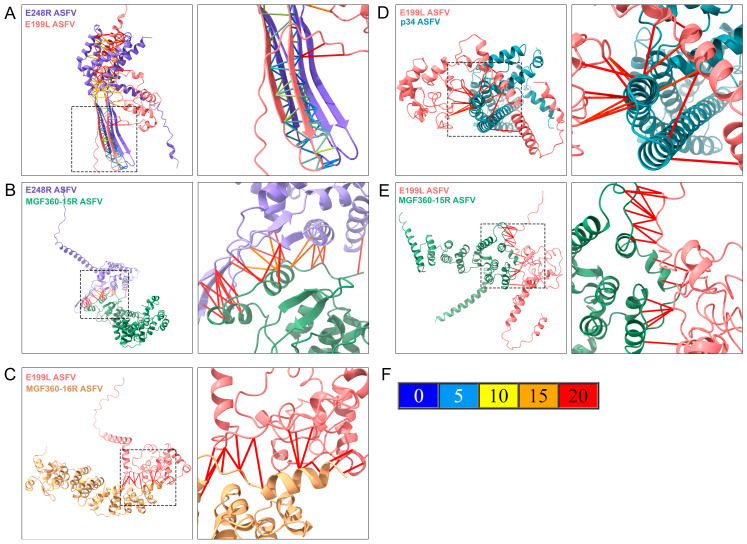
Characterization of potential protein–protein interactions between ASFV proteins E248R, E199L, p34, MGF360-16R, and MGF360-15R. ASFV proteins are shown in a multimeric state using one subunit of each protein modeled with AlphaFold2 (AF2) and displayed with the Chimera X visualization program. The first image of each figure represents the proteins in a multimeric display, showing alpha helices and beta sheets, where the image zoom is shown as a discontinued square. Each protein is colored by the same color identity used in Supplementary Figure S7 (purple for E248R, pink for E199L, green for MGF360-15R, blue for p34, and yellow for MGF360-16R). The amino acid interactions are shown in the second image due to AF2 structure prediction. AF2 predicted aligned error (PAE) colors the bonds between proteins, depending on the positional distance variation in Armstrong (Å). (**A**) Multimeric structure of E248R and E199L ASFV proteins. (**B**) Multimeric structure of E248R and MGF360-15R ASFV proteins. (**C**) Multimeric structure of E199L and MGF360-16R ASFV proteins. (**D**) Multimeric structure of E199L and p34 ASFV proteins. (**E**) Multimeric structure of E199L and MGF360-15R ASFV proteins. (**F**) Represents the predicted aligned error of AF2 (PAE), where the potential different distance variations in Armstrong are related to the protein-predicted bond colors.

Following immunoprecipitation assays, we selected proteins with robust elution expression patterns for a more detailed interaction model using the AlphaFold2 program. The interactions between ASFV proteins are visually represented as red lines in the immunoprecipitation scheme (Figure 6D). For the deeper interaction model, AF2 was employed in a multimeric display, modeling pairs of proteins as a multimer composed of one unit of each VP. Specifically, we modeled the interactions between the VP pairs E248R-E199L, E248R-MGF360-15R, E199L-MGF360-15R, E199L-MGF360-16R, and E199L-p34. To ensure accuracy, we selected the top-ranked model from 25 different AF2 models for each interaction. Two images of each multimer are presented in Figure 7. The first image illustrates the overall interaction between the two proteins, showing α helices and βsheets using the Chimera X program. A dot square emphasizes the position for the subsequent zoom image, where the protein interaction is most confidently predicted to occur.

The second image zooms in on the molecular interaction surface between the two proteins, providing a closer look at the predicted interaction details. The contact lines in Figure 7F, colored according to the AF2 PAE palette (Figure 7F), depict the variation in amino acid positional distances for the model. In the multimeric interaction model of entry-fusion ASFV proteins E248R and E199L, a highly accurate binding (from 0 to 5 Å of PAE) was observed between β strands of both proteins, indicating the presence of a highly stable domain for this interaction (Figure 7A). Stable positional predictions were also evident between E248R and MGF360-15R, with binding occurring between α helices and β sheets of both proteins (Figure 7B).

The potential interaction between E199L and MGF360-16R proteins could also involve α helices, or flexible regions of both proteins (Figure 7C). In the case of p34, a small region could interact with multiple regions of E199L, leading to a potential interaction between the proteins (Figure 7D). Additionally, the best multimeric display for E199L and MGF360-16R involved flexible regions of both proteins, resulting in an interaction with a potential 20 Å variation in the computational prediction by AF2 (Figure 7E).

For a comprehensive view, interaction models were visualized with the bFactor color of the complete structure (Supplementary Figure S7). Each protein in the multimer structure was easily recognizable by color and pLDDT score, highlighting high-confidence structures (blue regions) along the multimer structures.

## 4. Discussion

African swine fever (ASF) stands as an economically significant disease affecting both domestic swine and wild boar, lacking commercial treatments or vaccines. Infectious entry of ASFV hinges on the trafficking of mature ASFV virions through the endocytic pathway, in a coordinated multistep process dictated by several molecular signals, inherent in endosomes. This mechanism is still not completely understood, including information on virus and host proteins involved in these sequential stages.

In fact, viral decapsidation begins with an acidification-dependent step in the late endosome (LE), where the virion exposes its internal membrane, previous to fusion with the LE-limiting membrane [37]. The endosomal receptor NPC1 (Nieman Pick type C1), which is central in cholesterol flux at this level, is crucial for this fusion process [10]. ASFV internal membrane proteins E248R and E199L have been identified as direct interactors with the NPC1 receptor, being crucial in mediating viral fusion [10,12]. Notably, these proteins exhibit a high degree of conservation between various ASFV isolates, and demonstrated both homology and potential conserved structural motifs concerning the closely related VACV entry-fusion complex (EFC) proteins [10]. An illustrative example of this conservation is the presence of conserved cysteines distributed along the N-terminal region, a feature previously reported [10].

Specifically, ASFV protein E248R shares structural similarities with the L1R VACV protein, while ASFV E199L shows resemblances with VACV proteins A16, A26, and G9 [38]. It is noteworthy that VACV, which is a poxvirus and shares functional characteristics with the NCLDV group, has a common ancestral origin with ASFV [6]. The identification of common structural motifs, which are highly conserved in several members of this large viral family, suggests the potential functional importance of these proteins [39].

This shared taxonomy and structural similarity between ASFV and VACV proteins underscore the critical need to fill the existing knowledge gap concerning viral fusion and infection processes. We then performed whole-genome alignments to identify proteins in the ASFV genome that have similarities with the VACV EFC.

In this investigation, the initial selection of *Amsacta moorei* Entomopoxvirus (AmEPV) as the closest poxvirus to the *Asfarviridae* family was consistently supported by phylogenetic trees based on DNA polymerases and other proteins as presented in this manuscript and earlier studies [5,40,41]. Also, Faustoviruses was a close NCLDV family to ASFV in phylogenetic analysis from previous studies [42]. Therefore, these findings establish the way for extrapolating the results to this previously unexplored viral family.

Belonging to the Betaentomopoxvirus, AmEPV stands out as the sole subfamily within *Poxviridae* known to infect arthropods—a trait shared with ASFV, which can infect ticks as its arthropod host [41,43]. The fully sequenced genome AmEPV spans 32,392 base pairs (bp), with 294 open reading frames (ORFs) and featuring inverted terminal repeats (ITRs) [43]. Earlier studies, particularly those focused on thymidine kinase (TK) homology, have found the AmEPV TK gene 31.4% homology to the TK gene of ASFV. Notably, both ASFV and AmEPV exhibit the capacity for replication within arthropod hosts [44]. This strategic selection of a closely related poxvirus, recognized as a convergence member between ASFV and VACV genomes [44], allowed us to explore potential homologous proteins in the ASFV genome, providing valuable information concerning the relationships between these viruses.

Staggered genome alignments were performed between the VACV proteins constituting the entry-fusion complex and the complete genome of AmEPV, identifying the nine potential protein homolog candidates listed in Table 2. Subsequently, a homology search for these nine AmEPV proteins was carried out against the ASFV complete genome, obtaining the protein candidates summarized in Table 2. Notably, the ASFV entry-fusion proteins E248R and E199L exhibited similarities to VACV EFC proteins L1R, and proteins A16L, G9R, and A26L, respectively, corroborating recent studies [10,12]. Certain VACV proteins such as G3L, O3L, and A26L were absent in AmEPV, a trend supported by recent NCLDV studies [24]. In these instances, the alignment was directly made against the entire genome of ASFV, seeking potential similarities, not necessarily homologous proteins. However, these three genes seem to be important in the *Chordopoxviridae* subfamily due to their consistent conservation in their genome and their appearance after divergence from the crocodilepox virus. It could be reasonable to expect the presence of similar genes in ASFV, as they are able to infect mammals, as in the *Chordopoxviridae* subfamilies, where those three genes emerge [24].

Linear structure alignments between similar ASFV and VACV EFC proteins, facilitated the identification of similar amino acid regions, revealing related functional domain properties (shown in Supplementary Figure S2, gray lines). Additionally, identical amino acids shared in both proteins are highlighted in red, and similar amino acids, specifically, those sharing over 80% physicochemical amino acid properties, are marked in light red. These similar domains were expected to be exposed at the protein surface, allowing interaction with similar molecules with potentially analogous functional properties. To elucidate predicted structures, 3D protein structural models were generated using the AlphaFold2 (AF2) computational program [45,46], allowing visualization of potentially interacting residues (Supplementary Figure S3 and Figure 2). The observed structural similarities between VACV and ASFV proteins suggested important conserved roles for these proteins during infection [47].

Recent studies have highlighted the importance of cysteine-rich proteins and putative disulfide bonding patterns conserved in most poxviral species. In this study, we analyzed the conservation of disulfide bonds along target ASFV and VACV proteins. The E248R protein, previously described as a fusion protein, conserved cysteine sites, allowing the formation of two of the three disulfide bonds present in the L1R VACV homolog [10]. Conservation of disulfide bonds was also observed in VP E199L, among VACV homologs. The present study revealed potential disulfide bonds that could be conserved in its structure, based on the potential cysteines that could interact based on the AF2 models. This intramolecular disulfide bonding has been previously associated with an evolutionarily conserved entry-fusion mechanism for NCLDV entry [24], a hypothesis also proposed for ASFV. The superposition of these structures also revealed a structure spatial distribution conservation between these disulfide bond-conserved regions of VACV- and ASFV-selected proteins.

Interactions among proteins from the VACV entry-fusion complex have been recently characterized using proximity assays, immunoprecipitations, and in some cases, chemical cross-linking experiments, to characterize interactions between the proteins of the complex [18]. Immunoprecipitation was used in this study to explore the potential existence of an ASFV protein interaction network similar to that of VACV. Immunoprecipitation, a technique employed successfully with other highly infectious viruses [48], revealed positive interactions between fusion VP E199L and ASFV proteins MGF360-15R, MGF360-16R, MGF360-2L, p34 (CP2475L), M448R, and F165R. E199L also had a positive interaction with E248R. All these proteins were GFP-tagged except for E248R in which HA protein tag was used. No interaction was detected against the GFP protein control and another unrelated early viral protein I215L used as control. Given the potential functionality of the MGF families of ASFV, future research should delve into these polyvalent proteins that could interact with the viral or cellular proteins at different stages of the infection. These results suggest a novel interaction of these proteins potentially forming an entry-fusion complex similar to that of poxvirus, crucial in a membrane-fusion step. Additionally, our previous studies suggested that potential members of this complex share similar cellular targets in pairs, as described for E199L/p34 and E248R/MGF360-15R, suggesting common functions. Interestingly, the ASFV proteins E248R, E199L, and MGF360-15R share multiple cellular targets, particularly concerning Rho GTPases signaling and other cellular pathways [23]. The VPs G1340L, p34 (CP2475L), P1192R, M448R, E248R, and E199L have been found among the five structural domains of the ASFV particle by different techniques so far [49]. Furthermore, ASFV proteins E248R and E199L have been positioned at the fusion stage of the viral infection [10], and in this study, they were found to immunoprecipitate together with a strong signal. The interaction modeling revealed a low potential atomic distance error (PAE), between 0 and 5 Å for highly accurate models for each interaction, selected and analyzed with the ChimeraX program. These analyses have become increasingly important for understanding protein interactions and competitive binding models that provide highly accurate results [50,51].

Potential ankyrin repeats were detected in the MGF proteins 15R, 16R, and 2L. These domains consist of alpha helix-repeated regions packed in a nearly linear array linked with flexible loops, and have been widely described as mediators of protein–protein interactions [52]. These ankyrin repeats could mediate interactions with cellular targets in several infections [53].

Similarly, ASFV VPs p34 (CP2475L) and MGF360-15R were identified as potential ASFV entry-fusion proteins of the ASFV alongside E248R and E199L. Interactome analysis of these ASFV fusion proteins highlighted potential functions in the regulation of ASFV endosomal traffic, membrane fusion, and exit, sharing multiple cellular targets [23]. This suggests an orchestrated viral control of cellular processes through multiple VP interactions providing valuable insights into the ASFV infectious cycle.

## Figures and Tables

**Table 1:** NCBI reference sequences used for the *Poxviridae* and *Asfarviridae* phylogenetic tree. Poxviral and ASFV species were selected for the phylogenetic tree analysis from the NCBI website “https://www.ncbi.nlm.nih.gov/” (accessed on 20 January 2024). Family name, species, and NCBI reference sequence are shown in the table.

Family	Species	NCBI Reference Sequence
Poxviridae	*Bovine papular* Stomatitis virus	NC_005337.1
Poxviridae	*Amsacta moorei* Entomopoxvirus	NC_002520.1
Poxviridae	*Choristoneura rosaceana* Entomopoxvirus	NC_021249.1
Poxviridae	Eastern grey kangaroopox virus strain Sunshine Coast	NC_055229.1
Poxviridae	Eptesipox virus strain Washington	NC_035460.1
Poxviridae	Fowlpox virus	NC_002188.1
Poxviridae	Lumpy skin disease virus	OM984485.1
Poxviridae	*Molluscum contagiosum* virus subtype 1	NC_001731.1
Poxviridae	*Mythimna separata* Entomopoxvirus ‘L’	NC_021246.1
Poxviridae	Myxoma virus	NC_001132.2
Poxviridae	Nile crocodilepox virus	NC_008030.1
Poxviridae	Penguinpox virus isolate PSan92	NC_024446.1
Poxviridae	Sea otter poxvirus strain ELK	NC_037656.1
Poxviridae	Sheeppox virus 17077-99	NC_004002.1
Poxviridae	Vaccinia virus Western Reserve (WR)	NC_006998.1
Poxviridae	Variola virus	NC_001611.1
Asfarviridae	African swine fever virus isolate Georgia 2007/1	NC_044959.2
Asfarviridae	African swine fever virus isolate BA71V	NC_001659.2

**Table 2:** VACV EFC-, AmEPV-, and ASFV-similar protein gene names are shown in this table.

VACV EFC Proteins	AmEPV-Similar Proteins	ASFV-Similar Proteins
L1R	AMV217	E248R
F9L	AMV243	G1340L
A16L	AMV118	E199L
G9R	AMV035	E199L
H2R	AMV127	p34
A28L	AMV186	MGF365-15R
A21L	AMV249	MGF360-16R
L5R	AMV083	M448R
J5L	AMV232	MGF360-2L
G3L	- *	F165R
O3L	-	P1192L
A26L	-	E199L

* Not found in AmEPV whole genome. VACV EFC proteins were aligned in this case against the whole African swine fever virus genome to obtain ASFV target proteins.

**Table 3:** AlphaFold2 model prediction values. These serve as a metric defining the confidence of the predicted model for each protein. A score of 100 indicates the highest level of confidence, signifying a robust prediction of the spatial distribution of the protein’s amino acids within its structure. On the other end of the spectrum, a score of 0 suggests a model devoid of any confidence regarding the spatial arrangement of the protein’s amino acids.

	AlphaFold2 Model Confidence	Selected ASFV Proteins	AlphaFold2 Model Confidence
A21L	86.97	MGF360-2L	90.35
G9R	86.68	P1192L	89.13
H2R	85.75	MGF360-16R	87.95
A26L	85.51	M448R	87.23
G3L	84.80	G1340L	80.39
A16L	82.95	p34	75.99
F9L	82.93	MGF360-15R	64.45
A28L	81.59	E199L	61.38
J5L	80.92	E248R	43.20
L1R	78.85	F165R	40.40
L5R	78.53		
O3L	- *		

* Due to the low number of amino acids (35), AlphaFold2 does not provide a confidence value for the structure prediction model.

## Data Availability

The data presented in this study are available within the article and Supplementary Material.

## Supplementary Materials

The following supporting information can be downloaded at: https://www.mdpi.com/article/10.3390/v16030349/s1, Supplementary Table S1: Percentage of identity and similarity (identity/similarity) between VACV EFC proteins and AmEPV proteins; or VACV- and ASFV-similar proteins in pairs; Supplementary Figure S1: Amino acid alignments between VACV, AmEPV, and ASFV; Supplementary Figure S2: Protein alignments between VACV EFC proteins (top) and similar ASFV Georgia isolate proteins; Supplementary Figure S3: Structure prediction of VACV EFC- and ASFV-similar proteins with Alphafold2 program; Supplementary Figure S4: Transmembrane region prediction of ASFV VPs; Supplementary Figure S5: ASFV proteins conservation along different isolates from distances genotypes; Supplementary Figure S6: Immunoprecipitation assay triplicates; Supplementary Figure S7: AlphaFold bFactor representation of ASFV multimeric proteins E248R, E199L, p34, and MGF360-15R.
