# Supplementary material for: Identification of a Potential Entry-Fusion Complex Based on Sequence Homology of African Swine Fever and Vaccinia Virus
Source: Viruses. 2024 Feb 23;16(3):349. doi: 10.3390/v16030349 (PMC10975062; doi:10.3390/v16030349)
Supplement: Supplementary file 1 [file viruses-16-00349-s001.zip › viruses-2866724-supplementary.pdf]

Supplementary Paper Figures

Supplementary Table S1. Percentage of identity and similarity (identity/similarity) between VACV EFC proteins and AmEPV proteins; or VACV and ASFV similar proteins in pairs. The data obtained by Geneious Alignment following the procedure outlined in Materials and Methods section 2.3.

VACV EFC proteins	AmEPV selected proteins (identity/similarity against VACV EFC proteins in pairs)	ASFV selected proteins (identity/similarity against VACV EFC proteins in pairs)
L1R	AMV217 (32,4%/53,6%)	E248R (16%/31,1%)
F9L	AMV243 (28%/45,1%)	G1340L (6,7%/10,9%)
A16L	AMV118 (26,4%/43,8%)	E199L (13,3%/21,2%)
G9R	AMV035 (21,2%/40,5%)	E199L (15,9%/25,6%)
H2R	AMV127 (13,6%/29,3%)	P34 (13,8%/25,2%)
A28L	AMV186 (29,3%/50%)	MGF365-15R (14,5%/26,8%)
A21L	AMV249 (24,6%/42,4%)	MGF360-16R (9,6%/19,6%)
L5R	AMV083 (18,8%/35,6%)	M448R (8%/15,6%)
J5L	AMV232 (24,3%/49,3%)	MGF360-2L (11%/19,1%)
G3R	-	F165R (13,3%/25,2%)
O3L	-	P1192L (2,1%/2,5%)
A26L	-	E199L (11%/20,2%)

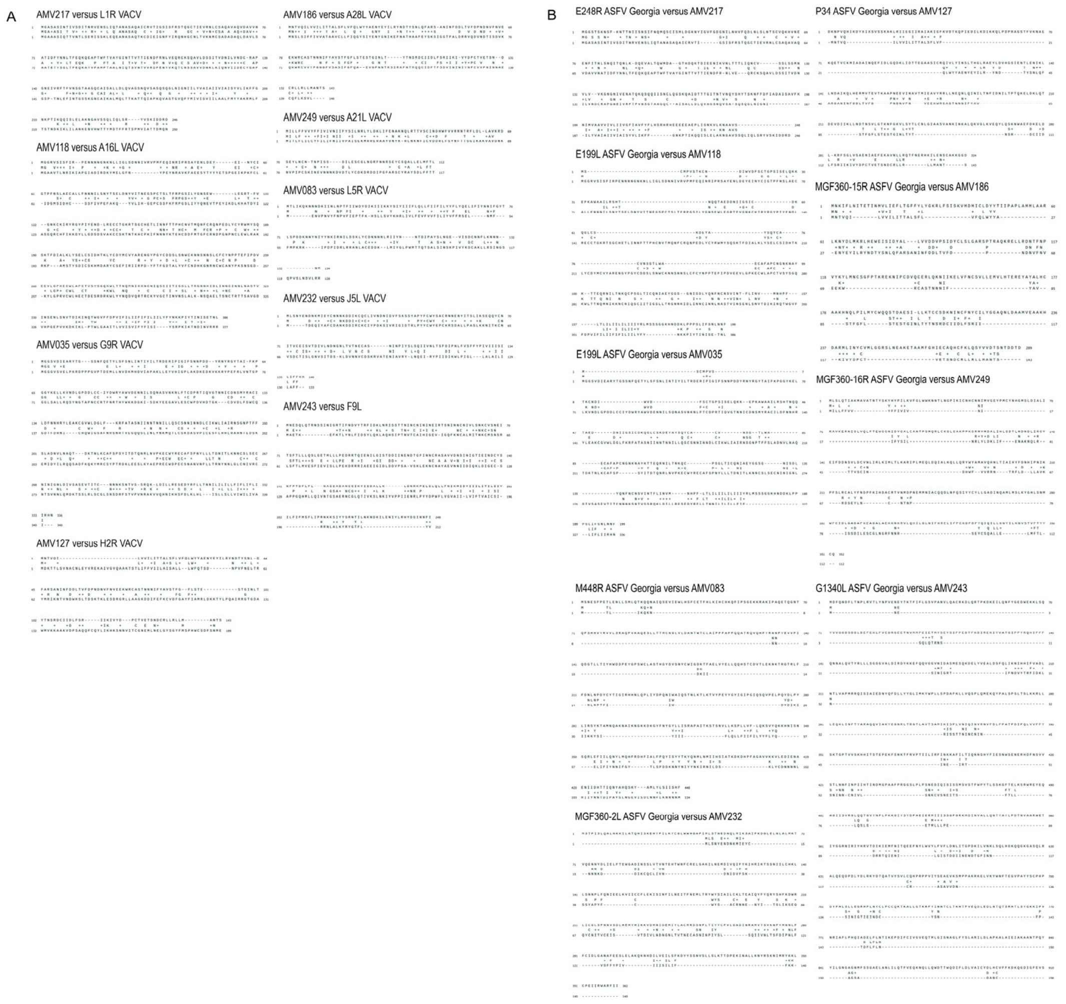

Supplementary Figure S1. Amino acid alignments between VACV, AmEPV, and ASFV. Figure 1A: Alignment between *Amsacta moorei* Entomopoxvirus (AmEPV) proteins (top) and their potential homologs in *Vaccinia* virus (VACV) EFC proteins. The alignment reveals the consensus amino acid sequences shared between the AmEPV and VACV proteins. Amino acid numbers are indicated along the sides of the sequences, facilitating easy identification and comparison. Similar amino acids are denoted by the symbol "+," highlighting regions where the amino acid residues are conserved or exhibit high similarity between the two virus species. **Figure 1B: Alignment between ASFV proteins (top) and their potential homologs in AmEPV proteins.** As in 1A.

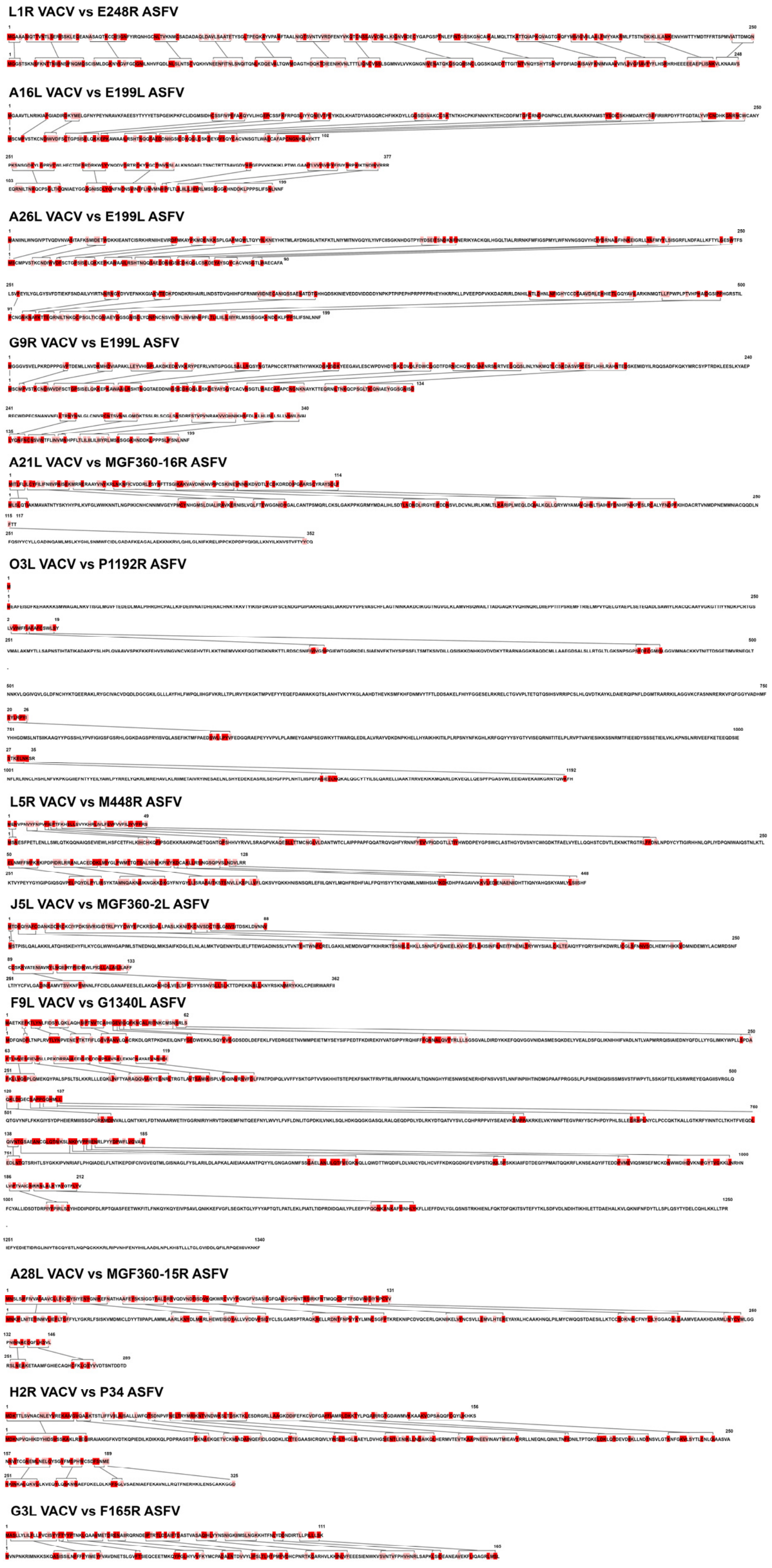

Supplementary Figure S2. Protein alignments between VACV EFC proteins (top) and similar ASFV Georgia isolate proteins. Each protein line is labeled with the amino acid number at its beginning and end. Identical amino acids are highlighted in red, emphasizing regions where the amino acid residues are

conserved between the VACV and ASFV proteins. Additionally, similar amino acids, defined as those with more than 80% similar physicochemical characteristics based on the Blosum62 matrix, are depicted in light red. To facilitate the visualization of the alignment and the comparison of similar protein domains, gray lines are used to connect corresponding regions in the alignment where similarity is observed.

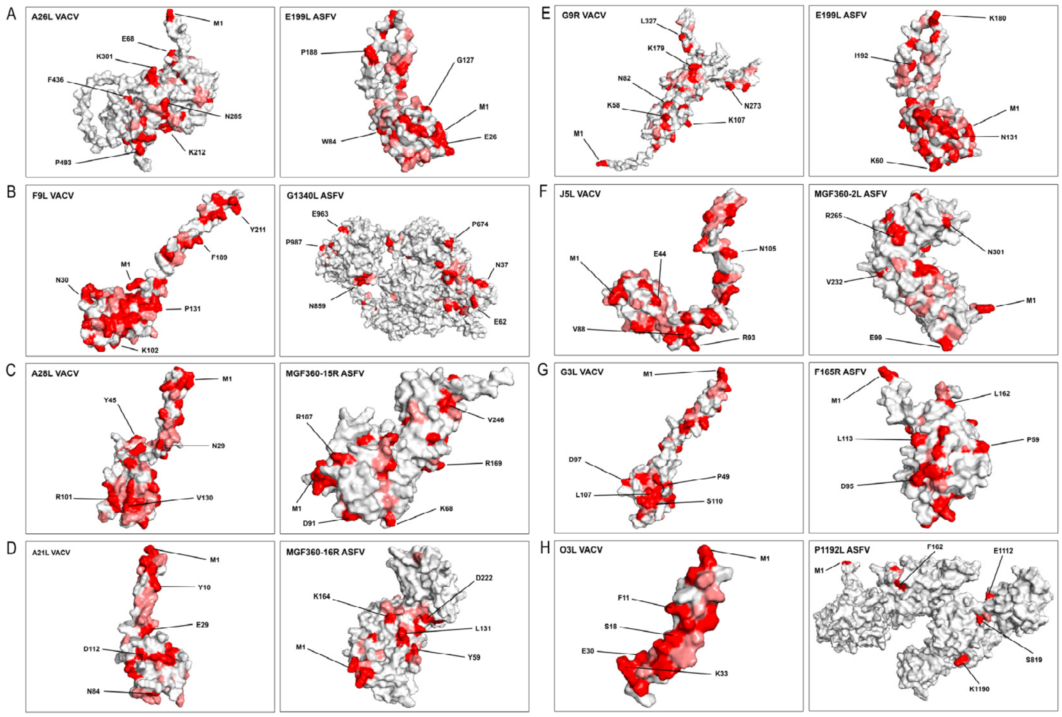

Supplementary Figure S3. Structure prediction of VACV EFC and ASFV similar proteins with Alphafold2 program. The 3D accurate computational model in this figure, generated using the PyMOL program, illustrates the structural comparison between VACV EFC proteins and similar ASFV proteins. The protein surfaces are displayed, with identical amino acids highlighted in red, forming pairs between VACV and ASFV proteins. Additionally, similar amino acids, characterized by more than 80% similarity in physicochemical characteristics according to the Blosum62 matrix, are depicted in light red. In each section of the figure, VACV EFC proteins are positioned on the left, while the corresponding ASFV similar proteins are on the right. Representative amino acids are numbered and indicated by lines in the protein models. The following pairs of protein homologs are featured: A) A26L of VACV and E199L of ASFV. B) F9L of VACV and G1340L of ASFV. C) A28L of VACV and MGF360-15R of ASFV. D) A21L of VACV and MGF360-16R of ASFV. E) G9R of VACV and E199L of ASFV. F) J5L of VACV and MGF360-2L of ASFV. G) G3L of VACV and F165R of ASFV. H) O3L of VACV and P1192R of ASFV.

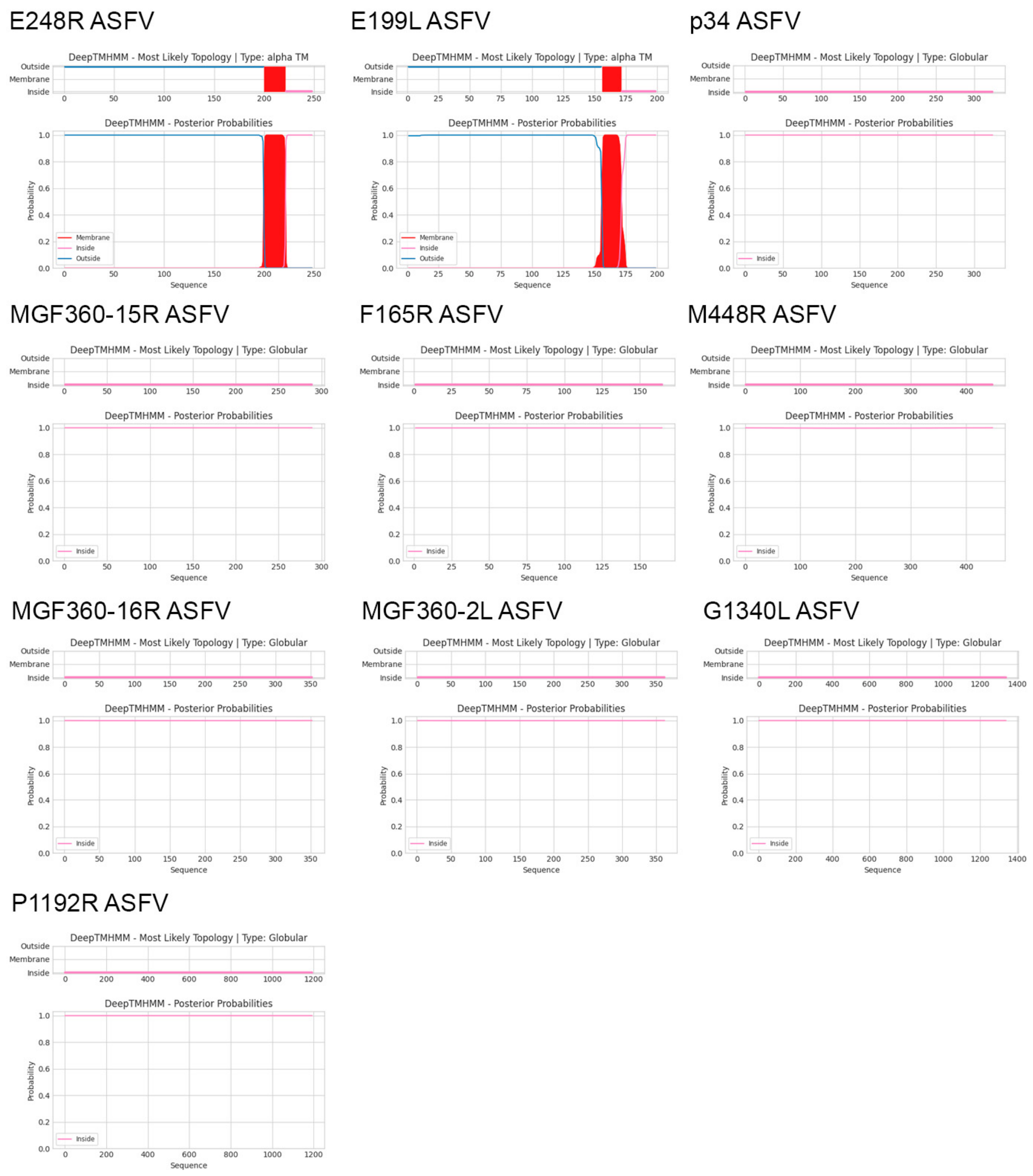

Supplementary Figure S4. Transmembrane region prediction of ASFV VPs. Transmembrane prediction of the viral proteins E248R, E199L, p34, MGF360-15R, F165R, M448R, MGF360-16R, MGF360-2L, G1340L, and P1192R. Each graphic generates the most accurate prediction for the topology of the proteins, represented by the sequence number of amino acids in both X axes. Two different graphics are represented for each protein regarding the Y axe: The first one shows the measurement of each domain (Outside, membrane domain, or inside of the membrane) in amino acids. The second graphic depicts the probability from 0 to 1 for each domain prediction. Blue line represents an outside protein domain, pink line is used for the inside domain, and red line for the transmembrane domain of the protein.

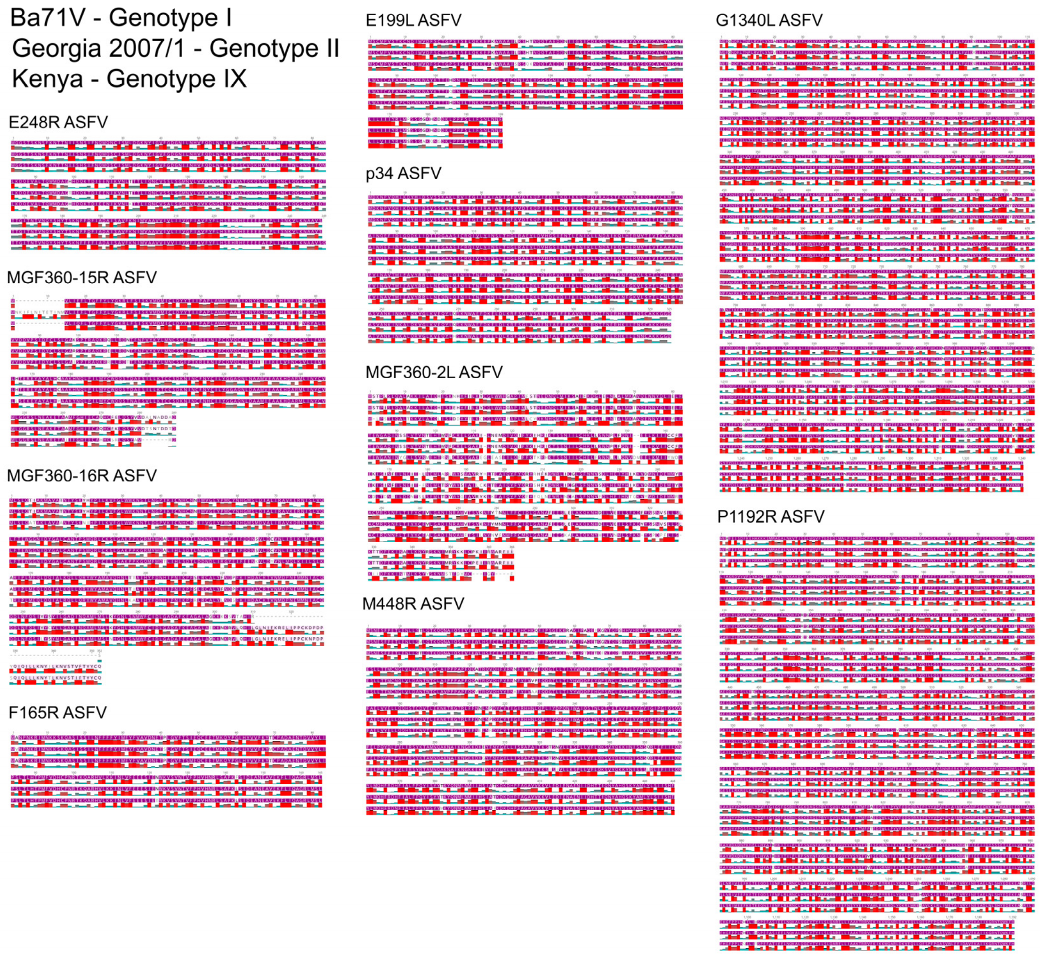

Supplementary Figure S5. ASFV proteins conservation along different isolates from distant genotypes. ASFV proteins from three different isolates and genotypes (Ba71V, Georgia 2007/1, Kenya) were aligned using the Geneious program using reference genomes following the procedure described in materials and Methods section 2.3. Ba71V ASFV from genotype I is represented in the first line of the alignments, Georgia 2007/1 isolate from genotype II in the second, and Kenya, from genotype IX in the third. Amino acid numbers are indicated at the top of each 10 amino acids. Purple color represents identical amino acids in three isolates, light pink color amino acids with more than 80% similar physicochemical characteristics, and white color with less than 80% physicochemical characteristics. Hydrophobic amino acids are shown in red and polar amino acids in blue. Gaps in the alignment are represented by -.

E248R HA cotransfected with ASFV EGFP and FLAG proteins

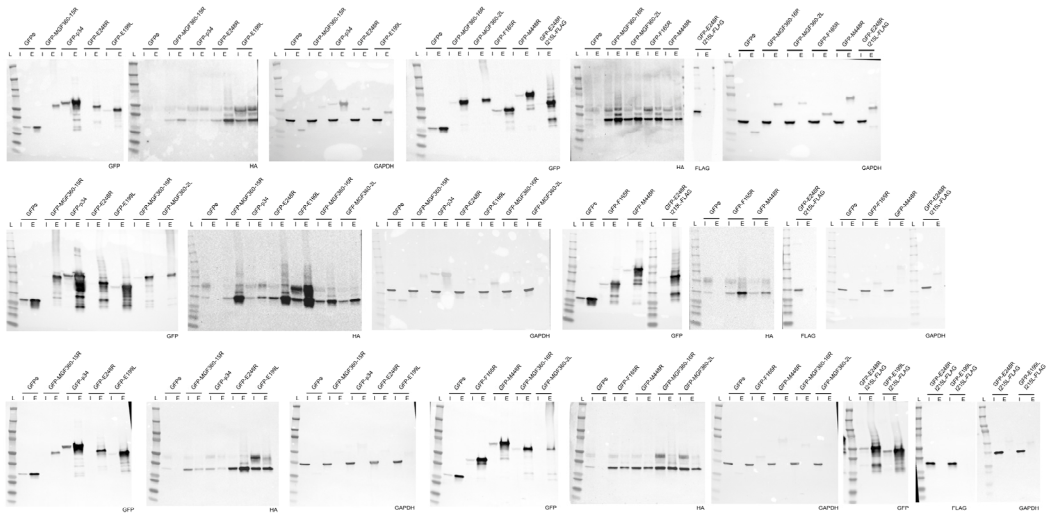

E199L HA cotransfected with ASFV EGFP and FLAG proteins

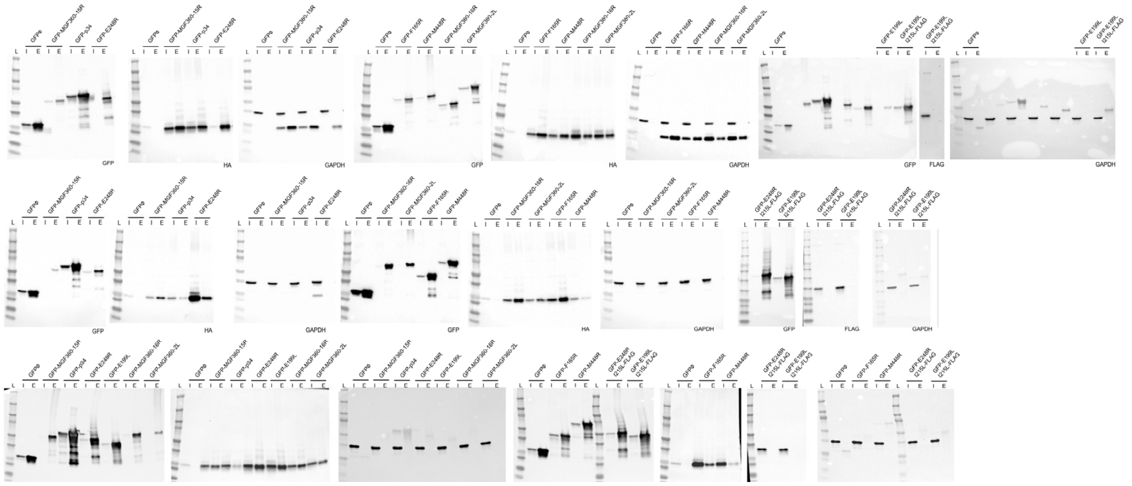

Supplementary Figure S6. Immunoprecipitation assay triplicates. Triplicates of the immunoprecipitation between HA-E248R and HA-E199L against GFP tagged proteins MGF360-15R, p34, E248R, E199L, MGF360-16R, MGF360-2L, F165R and M448R and ASFV I215L-FLAG protein.

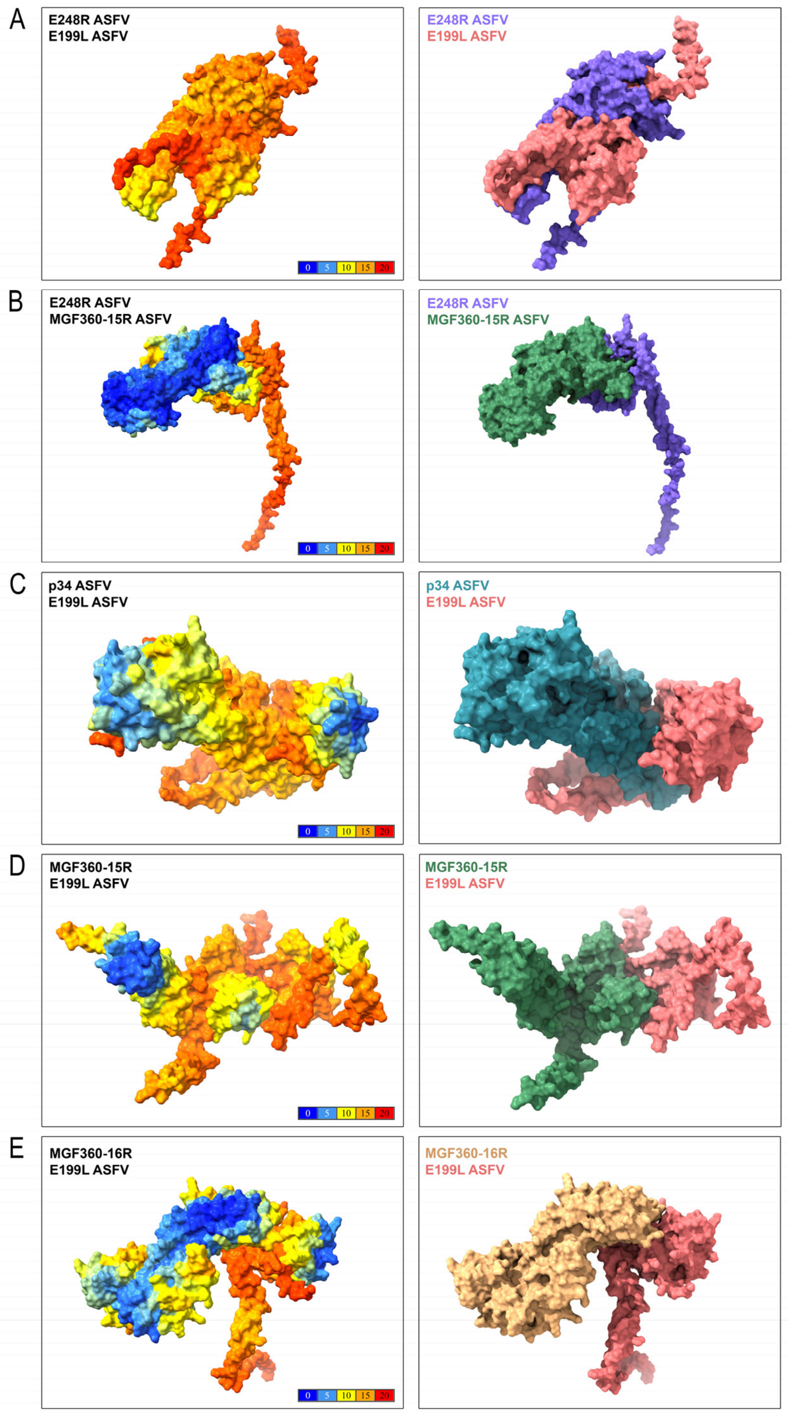

Supplementary Figure S7. AlphaFold2 bFactor representation of ASFV multimeric proteins E248R, E199L, p34, and MGF360-15R. Both images of each figure show the surface of the multimeric protein pair. The Alphafold bFactor pallet colors the surface of the first image protein structures (pLDDT score) of each figure. It is the blue color for a high confidence prediction value, yellow for a medium confidence prediction value, and red for a low confidence prediction value (high flexible structures of the proteins have more potential positions, and are the low confidence predicted regions), as shown in section 2.6 of materials and methods. Next to each bFactor figure, the same proteins, colored by protein identity are represented (E248R (purple), E199L (pink), MGF360-15R (green), p34 (blue), and MGF360-16R (yellow). The same color pallet is used in Figure 6.
